# Emotion Regulation is Associated with Increases in Linguistic Measures of Both Psychological Distancing and Abstractness

**DOI:** 10.1007/s42761-024-00269-7

**Published:** 2024-09-26

**Authors:** Erik C. Nook, Hayoung E. Ahn, Jessica L. Schleider, Leah H. Somerville

**Affiliations:** 1 Department of Psychology, Princeton University, Princeton, NJ USA; 2 Department of Psychology, Harvard University, Cambridge, MA USA; 3 Department of Medical Social Sciences, Northwestern University, Chicago, IL USA

**Keywords:** Language, Emotion regulation, Linguistic distance, Abstractness, Construal level theory

## Abstract

Emerging research shows that distancing one’s language by reducing the use of “I” and present-tense verbs is associated with more successful emotion regulation. However, construal level theory posits that increasing psychological distance should also shift mental representations to be more abstract. Here, we test whether successful emotion regulation is related to linguistic measures of both abstractness and psychological distance in two emotion regulation experiments reported previously (*N*_1_ = 217, *N*_2_ = 464). As hypothesized, we found that (i) linguistic measures of psychological distancing and abstractness were highly correlated, (ii) linguistic abstractness increased when people used cognitive reappraisal strategies to regulate their responses to negative images, (iii) instructing participants to distance their language by not using the word “I” increased linguistic abstractness, and (iv) the extent to which people made their language more abstract while regulating correlated with regulation success. These results extend theoretical arguments that psychological distance and abstract construals are strongly connected in the mind. Furthermore, they provide novel evidence that successful cognitive reappraisal is associated with more abstract representations of stimuli and that this cognitive shift can be achieved merely by shifting one’s language.

**Supplementary Information:**

The online version contains supplementary material available at 10.1007/s42761-024-00269-7.

Emotion regulation (i.e., the strategies people use to change their emotional experiences; Gross, 1998, 2015) is widely associated with mental health (Aldao et al., 2010; Gross & Jazaieri, 2014; Sloan et al., 2017). People can change how they feel by changing their thoughts, a process called *cognitive reappraisal* (Gross, 1998, 2015; McRae et al., 2012). One cognitive change people sometimes use is to shift their viewpoint, seeing a situation as further away from themselves in visual perspective, space, or time, a technique called *psychological distancing* (Kross & Ayduk, 2017). Meta-analyses show that shifting one’s perspective on negative memories from an “immersed” first-person focus to a “distanced” third-person focus reduces negative affect (Wallace-Hadrill & Kamboj, 2016). Similarly, imagining images as moving away from oneself (rather than towards oneself) or considering the impact of an event happening in the far future (rather than the near future) all downregulate negative emotions (Ahmed et al., 2018; Bruehlman-Senecal & Ayduk, 2015; Davis et al., 2011). Consequently, psychological distancing is seen as an effective emotion regulation strategy.

Interestingly, a growing body of research has shown that shifting the words people use (i.e., one’s language) can instill a sense of psychological distance and downregulate emotions. Specifically, instructing participants to stop using first-person singular pronouns and present-tense verbs downregulates negative emotions, ostensibly by increasing psychological distance from the self and the current moment (Nook, 2023; Orvell et al., 2019). For example, preparing for a stressful speech using “you” or one’s name rather than “I” reduces distress and improves performance (Kross et al., 2014), and writing without using first-person singular pronouns reduces affective reactions to aversive stimuli (Kross et al., 2017; Moser et al., 2017; Nook et al., 2017; Orvell et al., 2021). Thus, increasing *linguistic distance* can downregulate emotions. This relationship is also bidirectional, as several studies have shown that participants who more effectively use cognitive reappraisal spontaneously engage in more linguistic distancing while regulating (i.e., Nook et al., 2017; Nook, Vidal Bustamante et al., 2020; Shahane & Denny, 2019). As such, linguistic distance can provide a *measure* of emotion regulation efficacy. Finally, greater linguistic distancing correlates with less severe symptoms of anxiety and depression, as well as stronger response to psychotherapeutic interventions (Berry-Blunt et al., 2021; Cohen et al., 2022; Edwards & Holtzman, 2017; Nook et al., 2022). Thus, linguistic distance tracks both emotion regulation and mental health.

Construal level theory (CLT; Liberman & Trope, 2003, 2008) provides a framework for understanding how people distance their perspective and transcend their present situation. According to CLT, the mind collapses several “dimensions” of psychological distance onto a common mental ruler. Specifically, the mind represents physical distance (i.e., the distance between ourselves to physical objects or places), social distance (i.e., the proximity of our connections to others in a social network), temporal distance (i.e., the duration in time from the present moment to past or future times), and hypotheticality (i.e., whether things seem likely or unlikely) using a common code. Many studies now support this idea (Bar-Anan et al., 2006; Liberman & Trope, 2014; Maglio, 2020; Maglio et al., 2013). For example, the brain uses the same pattern of neural activity to represent shifts in stimuli along physical, social, or temporal dimensions (Parkinson et al., 2014). Additionally, people shift their perception of a stimulus on one distancing dimension if another dimension is altered (e.g., people think events happening geographically farther away are less likely than events close by, and people see themselves as less similar to their current self the further they project into the future; Wakslak, 2012; Wakslak et al., 2008). As such, the mind views these dimensions as interchangeable, inferring the distance on one dimension from the other. Crucially, shifting from close to distant perspectives is thought to arise by changing from concrete to abstract construals (Liberman & Trope, 2014). Per CLT, representing something as more psychologically distant increases its level of abstractness (i.e., conceptualizing it more in terms of its general properties rather than concrete details). Indeed, meta-analytic results show that increasing psychological distancing reliably increases measures of abstractness (Soderberg et al., 2015).

Interestingly, even linguistic evidence supports the notion that at least some distancing dimensions “travel together.” When people are instructed to stop using first-person singular pronouns (increasing distance along the “social” dimension), they spontaneously reduce their use of present-tense verbs (reflecting increased distance along the “temporal” dimension), and vice versa (Nook et al., 2017). An untested question is whether these shifts transcend social and temporal dimensions to also increase the abstractness of one’s representations. 

The current project addresses this gap using a linguistic measure of abstractness rooted in the linguistic category model (LCM; Semin & Fiedler, 1988, 1991). These researchers found that more abstract discussions were dominated by nouns and adjectives, whereas specific verb types characterized more concrete discussions. This prompted a purely linguistic measure of abstractness (called *linguistic abstractness*) that tracks human perceptions of abstractness (Nook, Stavish et al., 2020). Interestingly, LCM measures show that people use more abstract language when describing physically distant social events than proximal events (Fujita et al., 2006). Additionally, social media posts use more abstract language when discussing events farther away in space and time (Bhatia & Walasek, 2016; Snefjella & Kuperman, 2015). Finally, having people write about themselves without using the word “I” increases linguistic abstractness (Gainsburg & Kross, 2020). As such, there is preliminary evidence that linguistic distancing should also track linguistic abstractness, but the field has yet to extend this relationship to emotion regulation paradigms. Clarifying whether cognitive emotion regulation is related to increased abstractness can help identify the active ingredients of effective emotion regulation.

The current exploratory project seeks to test whether (i) emotion regulation is associated with increased linguistic abstractness and whether (ii) shifting linguistic distance along temporal or social dimensions also increases linguistic abstractness. To do so, we reanalyzed published studies (Nook et al., 2017) that examined the linguistic markers of effective emotion regulation (study 1) and tested whether instructing people to shift specific aspects of linguistic distance spontaneously reduces negative affect (study 2). We ask whether engaging in cognitive emotion regulation to downregulate negative affect increases linguistic abstractness and whether increasing linguistic distance also increases linguistic abstractness. Identifying abstractness as a key component of effective emotion regulation can guide future tactics for enhancing this crucial affective skill.

## Study 1

### Method

#### Participants

The current study involved additional analyses of data reported by Nook et al. (2017). Because results reported in that paper did not differ across study 1 (usable *N* = 107) and a replication sample of study 1 (usable *N* = 110; see Nook et al., 2017), we combined these subsamples to produce a full sample of *N* = 217 participants. Data from this combined sample were analyzed for the current report (age range = 19–71, *M* = 36.16, *SD* = 11.82, 139 [64%] female, 78 [36%] male). To participate in these online studies, participants were required to be located in the USA and have at least a 95% approval rating on previous online tasks. An additional 24 participants were collected in study 1 and its replication but were excluded due to noncompliance (see Nook et al., 2017 for details). We controlled for whether participants were collected in study 1 or its replication in all analyses presented here. Participants in these studies were recruited on Amazon Mechanical Turk (mTurk). Methods for both studies were approved by Harvard University’s Committee on the Use of Human Subjects, consistent with tenets of the Declaration of Helsinki. All participants for both studies provided informed consent prior to participating.

#### Stimuli and Procedure

Study 1 tested whether being instructed to regulate one’s emotions spontaneously increases the psychological distance of one’s language. Participants completed an adapted version of a classic cognitive reappraisal task (Nook et al., 2021; Ochsner et al., 2002, 2012). Participants saw 60 images that belonged to one of three conditions: *Look Negative* (negative images that they passively viewed), *Reappraise Negative* (negative images that they reinterpreted to reduce their negative emotional reactions), and *Look Neutral* (neutral images that they passively viewed). To assess the linguistic content of participants’ thoughts during each condition, participants transcribed their thoughts and feelings while viewing each image for 30 s. After each image advanced, participants were asked to rate their current negative affect by answering the question “How bad do you feel?” on a 7-point scale (1 = *Not bad at all* to 7 = *Extremely bad*). Images were drawn from normed image sets (e.g., the Open Affective Standardized Image Set (OASIS; Kurdi et al., 2017). To ensure differences across the *Look Negative* and *Reappraise Negative* conditions were due only to task instructions, two sets of images were selected so they were matched for valence, arousal, and image content, and list assignment to condition was counterbalanced across participants. Primary results replicated across studies, allowing us to combine samples for this set of analyses. See Nook et al. (2017) for additional methodological details.

#### Data Processing

We followed the methods of Mehl et al. (2012) to quantify the *linguistic distance* of participants’ language while responding to images in each condition. Participants’ text responses were processed using Pennebaker’s Linguistic Inquiry and Word Count (LIWC; Pennebaker et al., 2007) software to compute the percentage of words that fell within five categories: first-person singular pronouns (e.g., I, me, my), present-tense verbs, discrepancy words (e.g., would, could, should), articles (the, a, an), and words with more than six letters. More first-person singular pronouns, present-tense verbs, and discrepancies indicate more psychologically close language, whereas more articles and long words indicate more psychologically distanced language (Pennebaker & King, 1999). We *z*-scored the frequencies of all five categories across all trials for all participants, reverse-scored *z*-scores of the first three categories by multiplying them by − 1, and then averaged *z*-scores for all five classes together across trials within each condition for each participant. This produced a single measure for each participant of their linguistic distance in each condition, with higher values indicating greater linguistic separation from the here and now. Cronbach’s alpha for this measure was weak (*α* = .52), but see Table S1 in the Supplementary information for evidence that patterns of relations between individual variables behaved as expected (i.e., first-person pronoun and present-tense verbs are strongly related to each other; and first-person pronouns, present-tense verbs, and discrepancy words are inversely correlated with the other two measures).

We followed the methods of Seih et al. (2017) to compute the *linguistic abstractness* of participants’ language in each condition (which we verified does not use identical inputs as linguistic distancing, see below). This model uses a mathematical formula to compute abstractness scores for text entries by considering the proportion of words that fall within five different word classes and attaching weights to each word class according to which abstraction level it belongs to (see Carnaghi et al., 2008; Nook, Stavish et al., 2020; Semin & Fiedler, 1988, 1991 for details on the development, validation, and application of this approach). Abstraction level 1 includes descriptive action verbs, which describe a specific and observable action with a clear beginning and end (e.g., “walks”). Abstraction level 2 includes interpretive action verbs, which are used to characterize more general behavior that cross specific instances (e.g., “help”). Abstraction level 3 includes state verbs, which are used to describe emotional or mental states that do not contain a clear beginning and end (e.g., “admire”). Abstraction level 4 includes adjectives, which refer to abstract qualities and traits (e.g., “charming”). Abstraction level 5 includes nouns, which are considered to be the most abstract because they refer to classes of things (e.g., “athlete”). We used both Linguistic Inquiry and Word Count (LIWC) and TreeTagger software (Schmid, 1994; Seih et al., 2017) to count the number of words that fit into each level of abstraction, which were used to compute linguistic abstractness scores for each text response. Linguistic abstractness scores for all qualifying responses were then averaged within each condition for each participant. Scores ranged from 1 to 5, with higher values indicating higher levels of abstraction.

As in Nook et al. (2017), we computed measures of how much participants shifted their language while regulating, compared to responding naturally to negative images. Specifically, we computed a *Δ linguistic distancing* score for each participant by subtracting their average linguistic distancing score in the look negative condition from their average linguistic distancing score in the reappraise negative condition (Reappraise Negative–Look Negative). We computed parallel *Δ linguistic abstractness* scores by subtracting participants’ average linguistic abstractness score in the look negative condition from their reappraise negative average. More positive values for Δ linguistic distancing and Δ linguistic abstractness indicate that participants showed a larger increase in the use of abstract or distant language when instructed to regulate their emotions. Finally, we computed a measure of *reappraisal success* for each participant by subtracting their average negative affect rating for images in the reappraise negative condition from their average rating for images in the look negative condition (Look Negative–Reappraise Negative). Higher reappraisal success scores indicate that participants were more successful at reducing negative affect through cognitive reappraisal.

#### Ruling Out Circular Associations Between Linguistic Measures

It is possible that relations between linguistic distancing and linguistic abstractness emerge due to overlaps in scoring processes. If the same words are used to generate scores for both measures, then it is uninteresting to show an association between them because it emerges purely due to methodological overlap, rather than a deeper conceptual relationship between the constructs that these scores aim to measure. The inputs to the scoring systems described above are not identical, but to be sure, we demonstrated that one could engage in high linguistic distancing without shifting abstractness scores by showing that one could remove all first-person singular pronouns and present-tense verbs in a passage of text and not influence measures of linguistic abstractness. Specifically, we constructed two passages that were matched in all features except that they varied dramatically in linguistic distance (i.e., all first-person singular pronouns and present-tense verbs were removed) and found that shifting these key features of linguistic distance did not influence measures of linguistic abstractness. Both the *low distance* passage (“I am writing about how I feel. I am scared and I hate that the spider is so huge. This is terrible and I want it to stop. Why is this happening to me? I never want to see a bug like this again.”) and the *high distance* passage (“He was writing about how he felt. He was scared and he hated that the spider was so huge. This was terrible and he wanted it to stop. Why was this happening to him? He never wanted to see a bug like this again.”) produced identical linguistic abstractness scores of 2.8125, even though they would radically differ in *z*-scored linguistic distance scores. This demonstrates that the words important to the linguistic distancing measure (and those explicitly manipulated in study 2) do not influence the linguistic abstractness measure, ruling out a purely circular association between them.

#### Analyses

Analyses for both studies were exploratory in nature. We first sought to test whether linguistic measures of abstractness and distancing were significantly related to each other through a linear regression across the entire study, after controlling for sample (i.e., study 1 or study 1’s replication). There are minor differences between studies (i.e., different stimuli were used and participants differed), so we add these controls to adjust for any potential effect of these factors. We also tested for this association within each emotion regulation condition (Look Negative, Reappraise Negative, and Look Neutral). For a slightly stronger test of whether shifts in distancing tracked shifts in abstractness, we also tested for associations between Δ linguistic distancing and Δ linguistic abstractness. This analysis isolates a *person-level* effect specifically in the context of reappraisal, testing whether how strongly a person increases linguistic abstractness when regulating is related to how strongly they increase linguistic distancing when regulating. We hypothesized that these analyses would all result in significant positive associations.

We then examined whether participants’ language became more abstract when regulating emotions. We used a repeated-measures ANCOVA to test for significant differences in linguistic abstractness across the three emotion regulation conditions, after controlling for the study. Following a significant main effect of condition, we assessed for significant differences in linguistic abstractness between pairs of emotion regulation conditions (Reappraise Negative vs. Look Negative, Reappraise Negative vs. Look Neutral, Look Negative vs. Look Neutral). To control for the study, these follow-up pairwise comparisons were conducted within an ANCOVA framework with a type III sum of squares. We hypothesized that linguistic measures of abstractness would increase when participants engaged in cognitive reappraisal, as evidenced by higher linguistic abstractness in the reappraise negative condition compared to the look negative condition.

Finally, we tested whether the tendency to use more abstract language when regulating was associated with more successful emotion regulation. We used a linear regression to test the hypothesis that higher reappraisal success scores would be associated with increased linguistic abstractness, after controlling for study. We compared this result to a control analysis in which we tested whether “emotional reactivity” (i.e., the difference in self-reported negative affect between Look Neutral and Look Negative conditions; Silvers et al., 2012) was associated with the difference in linguistic abstractness between these conditions. If linguistic abstractness specifically tracks reappraisal efficacy and not just changes in negative affect, then we would hypothesize no relation between these two variables.

Preliminary analyses of research questions for study 1 and study 2 were conducted by the second author in 2018–2019. Final analyses and manuscript writing were completed by the first author in 2023–2024.

#### Software and Packages

Data were analyzed using R (version 4.3.0; R Core Team, 2023) implemented in RStudio (version 2023.6.1.524; Posit Team, 2023). For effect sizes of regression results, we report standardized betas and 95% confidence intervals (CIs) produced using the *standardize_parameters* function in the *effectsize* package (version 0.8.3; Ben-Shachar et al., 2020). ANCOVAs were conducted using the *ezANOVA* function in the *ez* package (version 4.4–0; Lawrence, 2016). For effect sizes of ANCOVA results, we report *η*_*p*_^2^ and 90% CIs produced using the *conf.limits.ncf* function in the *MBESS* package (version 4.9.2; Kelley, 2022). We present 90% CIs because *F* tests are one-sided (Lakens, 2013). Figures were produced using the *ggplot* function in the *ggplot2* package (version 3.4.2; Wickham, 2016) and the *ggarrange* function in the *ggpubr* package (version 0.6.0; Kassambara, 2023).

### Results

#### Task Effects

Participants reported higher negative affect in the Look Negative than the Reappraise Negative condition, and negative affect ratings were lowest in the Look Neutral condition (see Nook et al., 2017 for statistics). As such, participants overall reduced negative affect when reappraising negative images.

#### Relations Between Linguistic Abstractness and Linguistic Distancing

**Fig. 1:**
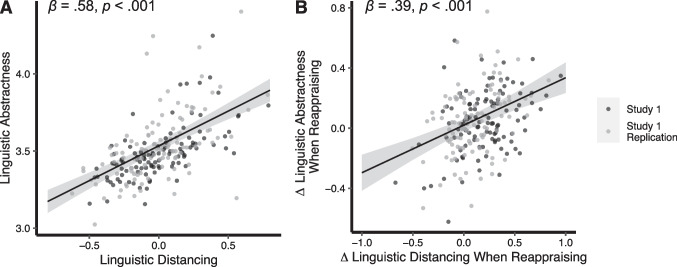
Study 1 linguistic abstractness and linguistic distancing results. **A** Scatterplot showing the correlation between average linguistic abstractness scores and average linguistic distancing scores, averaged across the three emotion regulation conditions. **B** Scatterplot showing the relation between Δ linguistic distancing (how much linguistic distancing increased when regulating) and Δ linguistic abstractness (how much linguistic abstractness increased when regulating). Gray-shaded regions represent 95% CIs

Linguistic abstractness scores and linguistic distancing scores showed a strong positive correlation, *β* = 0.58, 95% CI = [0.47, 0.69], *p* < .001 (Fig. 1a). The relationship between abstractness and distancing was present within each emotion regulation condition (Look Negative: *β* = 0.60, 95% CI = [0.49, 0.71], *p* < .001; Reappraise Negative: *β* = 0.46, 95% CI = [0.35, 0.58], *p* < .001; and Look Neutral: *β* = 0.55, 95% CI = [0.43, 0.66], *p* < .001). This means that when participants used more distant language, they also used more abstract language, in line with construal level theory. Additionally, Δ linguistic abstractness and Δ linguistic distancing showed a positive association, *β* = 0.39, 95% CI = [0.26, 0.52], *p* < .001, indicating that when participants reappraised a negative image rather than simply responding naturally, the extent to which they made their language more abstract was significantly correlated with the extent to which they made their language more distant (Fig. 1b).

#### Differences in Linguistic Abstractness Across Emotion Regulation Conditions

**Fig. 2:**
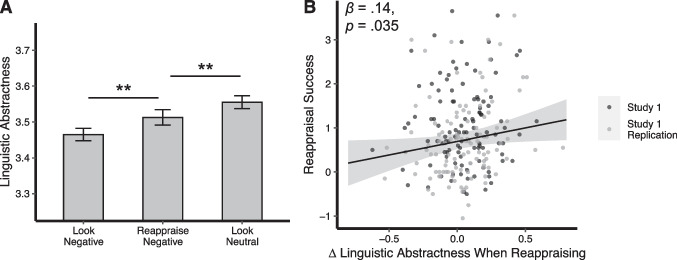
Study 1 linguistic abstractness results. **A** Average linguistic abstractness scores in each condition. Error bars represent 95% CIs, adjusted for within-person comparisons following (Morey, 2008). **B** Scatterplot showing the relation between reappraisal success (the extent to which participants reduced their negative affect when regulating) and Δ linguistic abstractness (how much linguistic abstractness increased when regulating). The gray-shaded region represents the 95% CI. **p < .01

Linguistic abstractness scores differed significantly across conditions, *F*(2, 432) = 21.78, *p* < .001, *η*_*p*_^2^ = 0.09, 90% CI = [0.05, 0.13] (Fig. 2a). Pairwise comparisons controlling for study revealed that linguistic abstractness was higher when writing about images in the reappraise negative condition (*M* = 3.51, *SD* = 0.23) than when writing about images in the look negative condition (*M* = 3.46, *SD* = 0.23), *F*(1, 216) = 11.00, *p* = .001, *η*_*p*_^2^ = 0.05, 90% CI = [0.01, 0.10]. Additionally, linguistic abstractness was higher in the look neutral condition (*M* = 3.56, *SD* = 0.24) than in both the reappraise negative condition, *F*(1, 216) = 8.35, *p* = .004, *η*_*p*_^2^ = 0.04, 90% CI = [0.01, 0.09], and the look negative condition, *F*(1, 216) = 58.87, *p* < .001, *η*_*p*_^2^ = 0.21, 90% CI = [0.14, 0.29]. Hence, participants used more abstract language when regulating their emotions. Interestingly, the condition with the highest abstractness was the Look Neutral condition. One explanation is that participants reported the least negative effect in this condition, meaning that people were most abstract when least distressed. It is also possible that language used for descriptions of the scenes used in this condition (e.g., colorful pencils) is naturally more abstract than scenes used in the other conditions (e.g., cars on fire). This result deserves further attention in future work.

#### Abstractness and Reappraisal Success

We observed a significant positive correlation between Δ linguistic abstractness and reappraisal success, *β* = 0.14, 95% CI = [0.01, 0.27], *p* = .035 (Fig. 2b). In other words, the extent to which participants reduced their negative affect ratings through reappraisal was significantly associated with the extent to which they increased their linguistic abstractness when they reappraised negative images. By contrast, the difference in self-reported negative affect between Look Negative and Look Neutral conditions (i.e., emotional reactivity) was not related to the difference in linguistic abstractness between these conditions, *β* =  − 0.04, 95% CI = [− 0.17, 0.10], *p* = .601. As such, shifts in linguistic abstractness seem to specifically track reappraisal success, not merely differences in negative affect between conditions.

#### Follow-Up Analysis Comparing Distancing and Abstractness

In this study, we have the opportunity to compare the strength of the relationships between reappraisal success and two linguistic measures: Δ linguistic abstractness and Δ linguistic distancing. We conducted two additional regressions to investigate this question. First, we tested for the strength of the relation between Δ linguistic distancing and reappraisal success after controlling for the study, finding a strong significant relationship, *β* = 0.28, 95% CI = [0.15, 0.41], *p* < .001. Notably, this effect size is double that of the relation between Δ linguistic abstractness and reappraisal success, reported above (i.e., *β* = 0.14). Interestingly, when adding both Δ linguistic distancing and Δ linguistic abstractness as predictors of reappraisal success (controlling for study), we find that Δ linguistic distancing remains a significant predictor, *β* = 0.27, 95% CI = [0.13, 0.41], *p* < .001, but Δ linguistic abstractness does not, *β* = 0.04, 95% CI = [− 0.09, 0.18], *p* = .542. We interpret this result in the “General Discussion” section.

## Study 2

The prior study showed that (i) linguistic measures of abstractness and psychological distance are strongly related, (ii) engaging in cognitive emotion regulation spontaneously increases the abstractness of language, and (iii) reappraisal success correlates with linguistic abstractness. Next, we provided a test of causality: Does manipulating linguistic distance increase linguistic abstractness?

### Method

#### Participants

Data from study 2 (usable *N* = 227) and the replication sample of study 2 (usable *N* = 237) from Nook et al. (2017) were combined to produce a full sample of *N* = 464 participants, as these samples produced statistically equivalent results (age range = 19–75 years, *M* = 35.96, *SD* = 12.04, 285 [61%] female, 178 [38%] male, 1 did not disclose gender). See Nook et al. (2017) for information on the 25 participants who were excluded due to noncompliance. All participants in these studies had not completed study 1. Again, to ensure that any slight differences between studies (i.e., different stimuli or participants) would not affect results, we controlled for whether participants were collected in study 2 or its replication in all analyses.

#### Stimuli and Procedure

Study 2 tested whether distancing one’s language spontaneously downregulates negative emotions. The study 1 paradigm was modified to assess whether shifting language from “psychologically close” words to “psychologically distant” words affects the emotional experience. Participants were randomly assigned to one of three between-subjects conditions: physical distance, social distance, and temporal distance. On each trial, they saw a cue word above an image and wrote their thoughts and feelings about the image for 30 s, and the cue words instructed them to use either psychologically “close” or “distant” language in their assigned distancing domain. For example, participants in the physical domain saw the cue word “HERE” above half of the images and “NOT HERE” above the other half, prompting them to write as if they were physically close or physically far away, respectively. Participants in the social domain condition saw the words “I” (write using the word “I”) or “NOT I” (write without using the word “I”), and participants in the temporal domain condition saw the words “NOW” (write using present-tense) or “NOT NOW” (write using a non-present tense) above images. Distance (close vs. far) was manipulated within subjects, and domain (physical, social, and temporal) was manipulated between subjects.

#### Data Processing

As in study 1, we proofread text entries from study 2 to remove spelling errors and then processed them using LIWC and TreeTagger. We computed each participant’s average linguistic distancing and linguistic abstractness scores as described above. Cronbach’s alpha for linguistic distance composite measure was stronger in this study (*α* = .73). See Table S2 in the Supplementary Information for correlations between each variable. It’s possible the alpha is higher in this study because two-thirds of participants were instructed to intentionally reduce one component of linguistic distancing in half of the trials (see below).

We computed Δ *linguistic distancing* in this study by subtracting participants’ average linguistic distancing score in the close condition from their average linguistic distancing score in the far condition (far–close). Similarly, we computed Δ *linguistic abstractness* by subtracting their average linguistic abstractness score in the close condition from that of the far condition (far–close). More positive values for Δ linguistic distancing or Δ linguistic abstractness indicated that participants showed a larger increase in the use of distant or abstract language when instructed to distance their language. Finally, we computed a measure of Δ *affect* for each participant by subtracting their average negative affect rating for images in the far condition from their average rating for images in the close condition (close–far).

#### Analyses

First, we sought to replicate the study 1 finding showing that linguistic abstractness correlates with linguistic distancing. We used a linear regression to assess whether participants’ overall average linguistic distancing and linguistic abstractness scores were significantly related after controlling for the sample. We also tested for this association across linguistic distancing conditions within each specific linguistic distancing domain (physical close, physical far, social close, social far, temporal close, and temporal far). Finally, we tested for associations between Δ linguistic distancing and Δ linguistic abstractness both in the overall sample as well as within each between-subjects distancing domain.

Next, we assessed whether manipulating linguistic distance impacted linguistic abstractness scores. We analyzed linguistic abstractness scores using a 2 [distance: close vs. far] × 3 [domain: physical, social, temporal] ANCOVA (controlling for the sample; i.e., study 2 vs. study 2 replication) with type III sums of squares. This tested whether there were significant differences in linguistic abstractness across linguistic distancing conditions and domains. When significant effects emerged, we examined whether each linguistic variable differed significantly between close and far instructions of each domain using ANCOVAs that again controlled for study. We hypothesized that abstractness scores would be higher in the far conditions compared to the close conditions, showing that intentionally distancing one’s language spontaneously makes one’s language more abstract. Additionally, this analysis allowed us to test whether the three domains of linguistic distancing varied in their impact on abstractness.

Finally, to assess potential relationships between abstract language and participants’ negative affect ratings, we tested for a relationship between Δ linguistic abstractness and Δ effect. In other words, we tested whether individuals who more strongly increased the abstractness of their language in the distancing conditions were also more successful at reducing their negative affect compared to individuals who had smaller shifts in linguistic abstractness. We used linear regression to test the hypothesis that Δ linguistic abstractness and Δ affect would be correlated (after controlling for the sample), suggesting that greater increases in linguistic abstractness were associated with improved affect ratings across the close and far conditions.

#### Transparency and Openness

See sections below for the link to the data and analytic code for this study. The software used for this study is identical to that used in study 1.

### Results

#### Relations Between Abstractness and Distancing

**Fig. 3:**
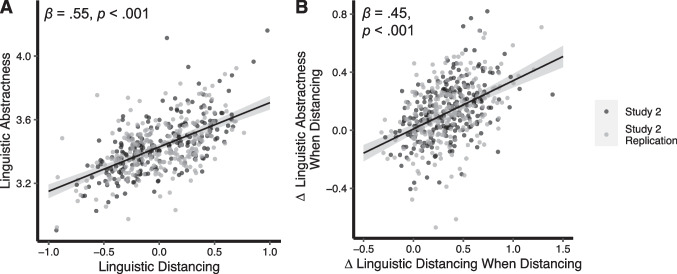
Study 2 linguistic abstractness and linguistic distancing results. **A** Scatterplot showing correlation between average linguistic abstractness scores and average linguistic distancing scores, collapsed across all conditions. **B** Scatterplot showing the relation between Δ linguistic distancing (how much linguistic distancing increased between close and far conditions) and Δ linguistic abstractness (how much linguistic abstractness increased when distancing). Gray-shaded regions represent the 95% CIs

Similar to study 1, we found a strong positive association between linguistic abstractness and linguistic distancing scores, *β* = 0.55, 95% CI = [0.47, 0.63], *p* < .001, in study 2 (Fig. 3a). This relationship was evident in each condition (physical close: *β* = 0.61, 95% CI = [0.48, 0.74], *p* < .001; physical far: *β* = 0.63, 95% CI = [0.51, 0.76], *p* < .001; social close: *β* = 0.41, 95% CI = [0.26, 0.56], *p* < .001; social far: *β* = 0.42, 95% CI = [0.28, 0.57], *p* < .001; temporal close: *β* = 0.61, 95% CI = [0.48, 0.73], *p* < .001; and temporal far: *β* = 0.52, 95% CI = [0.39, 0.65], *p* < .001). Additionally, the extent to which participants changed their level of distancing between close and far conditions (Δ linguistic distancing) was correlated with the extent to which they changed their level of abstractness (Δ linguistic abstractness), *β* = 0.45, 95% CI = [0.37, 0.53], *p* < .001 (Fig. 3b). This relationship was also evident within each domain (physical: *β* = 0.48, 95% CI = [0.34, 0.63], *p* < .001; social: *β* = 0.45, 95% CI = [0.31, 0.60], *p* < .001; and temporal: *β* = 0.32, 95% CI = [0.18, 0.28], *p* < .001). As such, individuals who more strongly increased their linguistic distance when instructed to do so also more strongly increased their linguistic abstractness.

#### Linguistic Abstractness During Distancing

**Fig. 4:**
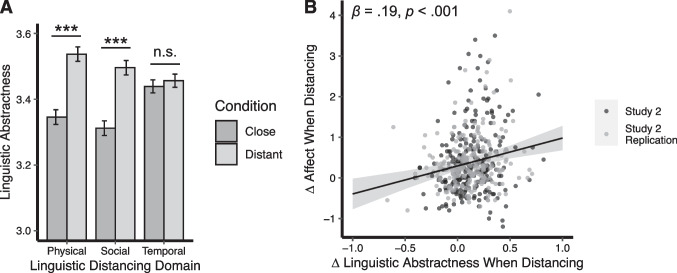
Study 2 linguistic abstractness results. **A** Average linguistic abstractness scores for close and far conditions within each distancing domain. Error bars are 95% CIs, adjusted for within-subjects comparisons following (Morey, 2008). **B** Scatterplot showing the relation between Δ affect (the extent to which participants reduced their negative affect when distancing) and Δ abstractness (how much linguistic abstractness increased when distancing). ***p < .001

Participants demonstrated increased linguistic abstractness when they distanced their language (e.g., not using the word “I”). As shown in Fig. 4a, linguistic abstractness scores differed significantly between close and far conditions, *F*(1, 461) = 216.13, *η*_*p*_^2^ = .32, 90% CI = [0.26, 0.37], *p* < .001. Participants showed higher linguistic abstractness scores when writing about images in the far condition (*M* = 3.50, *SD* = 0.20) than when writing about images in the close condition (*M* = 3.37, *SD* = 0.20). The main effect of domain was not significant, suggesting that linguistic abstractness did not differ significantly across the physical (*M* = 3.44, *SD* = 0.23), social (*M* = 3.40, *SD* = 0.21), and temporal (*M* = 3.45, *SD* = 0.20) domains, *F*(2, 461) = 2.90, *η*_*p*_^2^ = .01, 90% CI = [0, 0.03], *p* = 0.056. However, a significant interaction between distancing and domain emerged, suggesting that shifting from close to far language did not impact linguistic abstractness equivalently across domains, *F*(2, 461) = 42.23, *η*_*p*_^2^ = .15, 90% CI = [0.11, 0.20], *p* < .001.

Follow-up analyses investigated how linguistic abstractness varied across the close vs. far conditions of each distancing domain. Linguistic distancing was significantly lower in the physical close (*M* = 3.35, *SD* = 0.21) vs. the physical far condition (*M* = 3.54, *SD* = 0.21), *F*(1, 148) = 143.97, *η*_*p*_^2^ = .49, 90% CI = [0.40, 0.57], *p* < .001. Similarly, linguistic distancing was significantly lower in the social close (*M* = 3.31, *SD* = 0.18) vs. the social far condition (*M* = 3.50, *SD* = 0.19), *F*(1, 149) = 132.51, *η*_*p*_^2^ = .47, 90% CI = [0.38, 0.55], *p* < .001. However, linguistic distancing did not significantly differ between the temporal close (*M* = 3.44, *SD* = 0.19) vs. temporal far conditions (*M* = 3.46, *SD* = 0.21), *F*(1, 164) = 1.43, *η*_*p*_^2^ = .01, 90% CI = [0, 0.05], *p* = .233. Thus, instructing participants to distance their language by not using the word “I” (social distancing^1^) or by writing as if they were physically far away (physical distancing) increased linguistic abstractness. However, instructing participants to not use present-tense verbs (temporal distancing) did not affect linguistic abstractness.

#### Abstractness and Reduced Negative affect

We tested whether Δ linguistic abstractness correlated with Δ affect (how successful participants were in reducing negative affect through distancing). Linear regression revealed that the extent to which participants reduced their negative affect ratings through distancing was associated with the extent to which they increased their linguistic abstractness when distancing their language, *β* = 0.19, 95% CI = [0.10, 0.28], *p* < .001 (Fig. 4b).

## General Discussion

Here, we return to published studies that identified psychological distancing as a correlate of effective emotion regulation (Nook et al., 2017) to test an exploratory hypothesis rooted in construal level theory (Liberman & Trope, 2014) that psychological distancing should induce more abstract construals. In line with hypotheses, people spontaneously used more abstract language when regulating negative emotions, linguistic abstractness correlated with regulatory success, linguistic abstractness and linguistic distancing were strongly correlated with one another, and merely distancing aspects of one’s language (e.g., not using the word “I”) spontaneously increased linguistic abstractness. Together, these findings leverage psycholinguistic methods and the theoretical framework of CLT to extend our understanding of emotion regulation (see Table 1).

**Table 1:** Summary of study questions, results, and conclusions

Research question	Study 1 result	Study 2 result	Theoretical advance
Do linguistic measures of psychological distancing and abstractness correlate?	Linguistic distance and linguistic abstractness are strongly correlated (*β* = 0.58)	Linguistic distance and linguistic abstractness are strongly correlated (*β* = 0.55)	Results support the broad tenets of construal level theory using psycholinguistic measures
Does reappraising increase linguistic abstractness?	Linguistic abstractness significantly higher when reappraising negative images than responding naturally (*p* = 0.001)	N/A	Linguistic assessments suggest that cognitive reappraisal induces more abstract construals
Does increasing linguistic distance increase linguistic abstractness?	N/A	Linguistic abstractness is higher when participants are told to write as if images are further away in space or when told to not use the word “I” (*p*s < 0.001)	Provides causal support to the correlational finding above: Manipulating pronoun use increases the abstractness of language
Do people feel better the more they increase the abstractness of their language?	Linguistic abstractness significantly correlated with reappraisal success (*β* = 0.14)	Greater linguistic abstractness correlated with larger reductions in negative affect (*β* = 0.19)	Linguistic measures of abstractness can measure reappraisal success. Abstract construals may be an ingredient through which reappraisal reduces negative emotions

These results suggest that representing aversive scenes more abstractly may be a component of effectively reappraising them. Existing research suggests that abstract construals facilitate self-regulation and “wise” responding (Kross & Grossmann, 2012; Mischel & Rodriguez, 1993). However, to our knowledge, this is the first depiction of abstractness as a correlate of regulatory success in cognitive reappraisal tasks. This result stands to reason, as reappraisal by necessity involves thinking creatively to take a new perspective or imagine an alternative interpretation of the situation at hand. One could be concerned that the findings presented here emerge only because people in this task used a distancing strategy for regulating their emotions (e.g., imagining these aversive images from a third-person perspective or as happening far away). Fortunately, descriptive coding of reappraisals from this task in another sample revealed that participants used such an explicit distancing technique in only 4% of trials (Nook, Vidal Bustamante et al., 2020). Thus, abstractness seems to be a general quality of effective cognitive reappraisal even outside of the specific strategy of distancing one’s perspective.

However, the broader literature presents something of a puzzle as to whether abstractness is helpful or unhelpful in emotion regulation. On the one hand, therapies like cognitive behavioral therapy, the unified protocol, and dialectical behavior therapy (Barlow et al., 2011; Beck, 2011; Linehan, 1993) begin treatment by providing patients with an abstract model of how their thoughts, feelings, and behaviors interact to produce their symptoms, and these *psychoeducation* modules appear to benefit patients (Tursi et al., 2013). Thus, helping people (or at least clinical populations) take an abstract view of themselves seems to help them manage difficult emotions. On the other hand, a recent meta-analysis by Moran and Eyal (2022) showed that psychological distancing tasks reliably reduce negative affect in non-clinical samples, but increased abstractness only reduces the intensity of “low-level” emotions (e.g., more concrete basic emotions like sadness rather than more abstract self-conscious emotions like envy). In fact, worry and rumination (maladaptive cognitive processes) are characterized by *elevated* abstractness (Goldwin & Behar, 2012). Thus, it seems that the affective impact of abstraction can be helpful or unhelpful, depending on context. The current study adds an important data point to this ongoing discussion: When people use cognitive reappraisal to regulate their responses to aversive images, the use of more abstract language appears to track improved regulatory success. Whether this generalizes to other stimuli or contexts is an important question for future research.

These results also contribute to the CLT literature. As reviewed in the introduction, there is substantial evidence that dimensions of psychological distance are related and that increasing psychological distance increases the abstractness of one’s construal. However, evidence for a connection between distance and abstractness at the purely linguistic level is less established (see Fujita et al., 2006; Gainsburg & Kross, 2020). Here, we show strong, replicable correlations between linguistic measures of psychological distancing and abstractness, and study 2 established a causal connection between them. Nonetheless, an outstanding question is whether these shifts arise for purely “linguistic” reasons (e.g., the English language is structured such that not using “I” necessitates more abstract verb use) or for more “psycholinguistic” reasons (e.g., not using the word “I” induces psychological distance, which increases the abstractness of appraisals). Disentangling these possibilities requires a close examination of grammatical constructions in English. That said, even the former explanation is theoretically interesting, as it may be the case that the structure of English reflects a deep mental connection between distance and abstraction (Jackson et al., 2022).

Nonetheless, open questions remain. Even though LCM scores of linguistic abstractness have correlated with human-coded abstractness in other studies (Nook, Stavish et al., 2020; Semin & Fiedler, 1991), future research could substantiate this assumption in tasks like these and demonstrate that reappraisals are seen as more abstract than natural responses to aversive stimuli. If so, researchers could then test (i) whether merely inducing more abstract construals of aversive stimuli downregulates negative affect, (ii) the duration of these effects, and (iii) whether we see similar effects on more naturalistic or personally relevant sources of emotion (e.g., memories; Kross et al., 2009; St. Jacques et al., 2017). These approaches could establish the causal role of abstractness in emotion regulation and advance the ecological validity of the current findings. This seems especially important given the research summarized above showing that abstractness is not always associated with lower emotional intensity and may be moderated by context. Additionally, we found that Δ linguistic abstractness was a weaker correlate of reappraisal success than Δ linguistic distancing. It is possible that distancing one’s perspective without increasing abstractness is the most “active” ingredient in effective reappraisal. However, it is also possible that our linguistic operationalization of abstractness may be less closely tied to abstract cognition than linguistic distancing is to psychological distancing. If so, these results may reflect differences in measurement error, rather than a true difference in psychological processes. A more thorough investigation of the operationalizations of these constructs would provide a firmer test of the relative strengths of these two constructs.

In sum, we integrated psycholinguistics, CLT, and emotion regulation paradigms. Results affirm a coupling between distance and abstractness while also prompting new questions about when and how an abstract perspective facilitates emotion regulation.

## Electronic supplementary material

Below is the link to the electronic supplementary material.

Supplementary file1 (PDF 105 KB)

## Footnotes

1 The term *social distancing* here does not refer to the COVID-19 parlance of physically separating oneself from others. Instead, we mean it in the framework of construal level theory (i.e., adopting perspectives that increase the perceived distance between oneself and a stimulus).

## Additional Information

### Data Availability

Data for both studies are available on the Open Science Framework (https://osf.io/mvks2/).

### Code Availability

Analytic code for both studies are available on the Open Science Framework (https://osf.io/mvks2/).
