# Supplementary material for: Emotion Regulation is Associated with Increases in Linguistic Measures of Both Psychological Distancing and Abstractness
Source: Affect Sci. 2024 Sep 26;6(1):63–76. doi: 10.1007/s42761-024-00269-7 (PMC11904052; doi:10.1007/s42761-024-00269-7)
Supplement: Supplementary file 1 — Supplementary file1 (PDF 105 KB) [file 42761_2024_269_MOESM1_ESM.pdf]

Supplemental Materials for

Emotion regulation is associated with increases in linguistic measures of both psychological distancing and abstractness

Nook, Ahn, Schleider, & Somerville

Contents

I. Correlations between linguistic distancing components

I. Correlations between linguistic distancing components

We use a composite measure of linguistic distance established by prior work (Cohn et al., 2004; Mehl et al., 2012; Pennebaker & King, 1999). For those curious as to the strength and patterns of relations between individual components of this measure in the current datasets, we provide correlation tables below. Note that trial-level assessments of each linguistic component are nested within conditions, which are nested within individuals. Given that such nesting creates issues for correlation measures (which assume one observation per person), we have conducted correlations on averages of each variable for each participant. Thus, these values assess the extent to which people who are higher in one component tend to be high (or low) in the other components. Patterns resemble those presented in the original Pennebaker & King (1999) paper: (i) first-person pronoun and present-tense verbs are strongly related to each other and (ii) first-person pronouns, present-tense verbs, and discrepancy words are inversely correlated with the other two measures.

Table S1. Correlations between linguistic distancing subcomponents in Study 1

	1.	2.	3.	4.
1. First-person singular pronouns	-			
2. Present-tense verbs	$.62^{***}$	-		
3. Discrepancy words	$.21^{**}$	$.39^{***}$	-	
4. Articles	$-.18^{**}$	.05	$-.27^{***}$	-
5. Words > 6 letters	$-.24^{***}$	$-.60^{***}$	$-.25^{***}$	$-.37^{***}$

Note: Correlations are performed on person-level averages for each component, *** $p < .001$, ** $p < .01$

Table S2. Correlations between linguistic distancing subcomponents in Study 2

	1.	2.	3.	4.
1. First-person singular pronouns	-			
2. Present-tense verbs	$.53^{***}$	-		
3. Discrepancy words	$.45^{***}$	$.47^{***}$	-	
4. Articles	$-.51^{***}$	$-.56^{***}$	$-.56^{***}$	-
5. Words > 6 letters	$-.27^{***}$	$-.39^{***}$	$-.22^{***}$	< .01

Note: Correlations are performed on person-level averages for each component, *** $p < .001$
